# Exploring Public Awareness of Overwork Prevention With Big Data From Google Trends: Retrospective Analysis

**DOI:** 10.2196/18181

**Published:** 2020-06-26

**Authors:** Ro-Ting Lin, Yawen Cheng, Yan-Cheng Jiang

**Affiliations:** 1 Department of Occupational Safety and Health College of Public Health, China Medical University Taichung Taiwan; 2 Institute of Health Policy and Management, College of Public Health, National Taiwan University Taipei Taiwan

**Keywords:** overwork, working hours, policy, big data

## Abstract

**Background:**

To improve working conditions and prevent illness and deaths related to overwork, the Taiwanese government in 2015, 2016, and 2018 amended regulations regarding working time, overtime, shifts, and rest days. Such policy changes may lead to a rising public awareness of overwork-related issues, which may in turn reinforce policy development.

**Objective:**

This study aimed to investigate to what extent public awareness of overwork-related issues correlated with policy changes.

**Methods:**

Policies, laws, and regulations promulgated or amended in Taiwan between January 2004 and November 2019 were identified. We defined 3 working conditions (overwork, long working hours, and high job stress) related to overwork prevention, generated a keyword for each condition, and extracted the search volumes for each keyword on the Google search engine as proxy indicators of public awareness. We then calculated the monthly percentage change in the search volumes using the Joinpoint Regression Program.

**Results:**

Apparent peaks in search volumes were observed immediately after policy changes. Especially, policy changes in 2010 were followed by a remarkable peak in search volumes for both overwork and working hours, with the search volumes for overwork increased by 29% per month from June 2010 to March 2011. This increase was preceded by the implementation of new overwork recognition guidelines and media reports of several suspected overwork-related events. The search volumes for working hours also steadily increased, by 2% per month in September 2013 and afterward, reaching a peak in January 2017. The peak was likely due to the amendment to the Labor Standards Act, which called for “1 fixed and 1 flexible day off per week,” in 2016. The search volumes for job stress significantly increased (*P*=.026) but only by 0.4% per month since March 2013.

**Conclusions:**

Over the past 15 years, Taiwanese authorities have revised and implemented several policies to prevent overwork-related health problems. Our study suggests a relationship between the implementation of policies that clearly defined the criteria for overwork and working hours and the rising public awareness of the importance of overwork prevention and shorter working hours.

## Introduction

Overwork—including long hours or high physical or psychological effort or strain—may lead to the occurrence of cerebrovascular and cardiovascular diseases, mental disorders, and even *karoshi* (occupational sudden death) [1-3]. The first *karoshi* case was reported in Japan in 1969: the death of a 29-year-old male worker in a shipping department due to a stroke [4]. An increasing number of overwork-related events have been reported ever since in Japan, South Korea, and Taiwan, where the culture of long working hours is prevalent and even valued [5].

In Taiwan, several apparent overwork-related cases were reported in the period between 2009 and 2011 but their workers’ compensation claims were disapproved by the Bureau of Labor Insurance. The ways the labor insurance authorities handled such disputes ignited public criticisms. Finally, these workers’ family members turned to legislators for help, pushing the governments to revise their policies and regulations concerning overwork [6]. In response to societal concerns, the Taiwanese government has modified both the policies for workers’ compensation benefits and the prevention policies for overwork [6]. With regard to workers’ compensation, the government has loosened the recognition guidelines since the late 2010 to allow more cases to be compensated by the labor insurance system. With regard to prevention of overwork problems, the government adopted new prevention measures in the Occupational Safety and Health Act in 2013; revised legal regular working hour limit stipulated in the Labor Standards Act in 2015 from 84 hours over 2 weeks to 40 hours per week; and twice—in 2016 and again in 2018—amended regulations pertaining to regular working time, overtime, annual leave, shifts, and rest days. Through these actions, the government has demonstrated its commitment to preventing overwork by shortening working hours and improving working conditions.

In addition to regulatory reforms, for the prevention of overwork problems, it is important to enhance public awareness of health risks associated with overwork and other adverse working conditions, such as long working hours and high job stress [7,8]. On the one hand, policy changes may be the results of increased social concerns. On the other hand, the development of policies could further enhance social awareness. According to the knowledge, attitude, and practice theory [9], it can be expected that higher social awareness will lead to the acquisition of information and knowledge and the cultivation of better attitudes toward preventative practices. For example, clear regulation of a company’s working hours could facilitate better understanding of how to prevent overwork among its employees. However, public awareness levels are not easily assessed.

The use of internet and online search engines such as Google, Yahoo!, and Bing has become one of the most popular means to obtain knowledge on specific topics, which further enhance people’s attitudes, behaviors, and decisions [10]. A previous study has identified that in 2012, around 55% of internet users in the United States used search engines to obtain health-related information [11]. Among all search engines, Google is the most popular in many countries, and in Taiwan about 94% of internet users use Google as their primary search engine [12]. The Google Trends website reports on recorded Google search volumes, and has been widely used to measure public awareness and interest in specific issues, such as cancer screening and crude oil prices [13,14], to assess the impact of celebrity on public behavior [15], and to conduct health assessment studies [16]. Thus, Google search volumes extracted from the Google Trends platform can be a proxy indicator of public awareness of overwork-related issues.

Previous studies in Japan and South Korea have mentioned that public awareness of overwork is likely to be enhanced both by reports of the occurrence of overwork-related diseases and by recent changes in overwork-related policy [7,17]. While this assumption has been proposed, no study has yet demonstrated whether or not public awareness has been influenced by policy changes and reported overwork incidents. We hypothesized that public awareness increased following changes in policies which clearly address overwork-related risk factors. We therefore aimed to use Google search volumes to investigate whether and to what extent the public’s awareness of overwork prevention has been influenced by policy changes and the occurrence of overwork-related events.

## Methods

### Study Process

**Figure 1:**
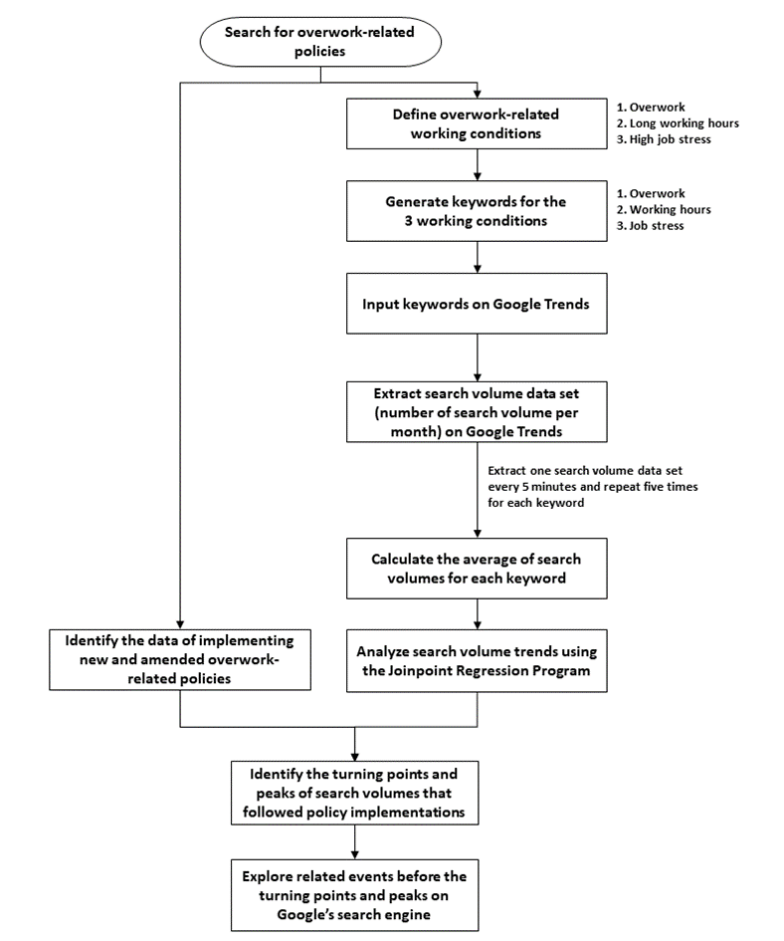
The study process.

Figure 1 shows our study process. We searched for policies, laws, and regulations promulgated or amended in Taiwan during the period between January 2004 and November 2019. We then identified 2 working conditions related to the aforementioned policy changes: (1) overwork, which has no official definition in Taiwan but is understood to describe both working too long (too many hours) and as working too hard (physically or psychologically); and (2) long working hours, which are regulated through both regular working time and overtime policies. We further identified a third working condition related to the 2004–2019 policy changes but had no direct quantitative measure: (3) high job stress. We treated overwork and long working hours as the main topics of interest, and high job stress as a reference topic (relevant to overwork but not addressed in the amendments of Labor Standards Act). To measure these 3 working conditions, we identified a keyword for each condition.

We used the keywords to extract search volumes from the Google Trends platform [18], which is used to record big data related to search volumes. The data on search volumes were scaled on a range of 0-100, with the scaled number shown as relative proportional to all searches. The website has 4 filter options: region, time, category, and search type; for these filter options, we selected Taiwan, January 2004–November 2019, all categories, and web search, respectively.

We noticed the variability of search volumes while we repeatedly searched on the Google Trends platform. The Google Trends sampled data on searches correspond to billions of Google searches per day. However, the algorithms behind the platform were not available to the general public [19]. We, therefore, developed our search strategy, repeated data extraction, and smoothed data by taking averages. We extracted one search volume data set every 5 minutes and repeated five times for each keyword. We then calculated the average of search volumes for each keyword and analyzed search volume trends using the Joinpoint Regression Program [20]. Next, we identified the turning points and peaks of search volumes that followed policy implementations. For points or peaks that did not follow policy changes, we used Google’s search engine to explore related events before the turning points and peaks.

### Study Period

Our study period was from January 2004 to November 2019. January 2004 was the earliest data we could access from the Google Trends platform, and 2004 was also the year when Taiwan’s Recognition Guidelines for Overwork-Related Diseases were first amended.

### Data Analysis

First, we calculated the average monthly search volume for each keyword. Second, we used the Joinpoint Regression Program to estimate the web search volume trends over time [20]. The Joinpoint Regression Program allows us to analyze trend data and test joinpoints (or apparent changes or peaks) across the whole study period. Data captured as 0 were adjusted to 0.5 to fit all data on a log-linear Poisson regression model. Bayesian information criterion was used to identify turning points and select the best model. The monthly percentage change of search volumes of each segment during the study period was estimated by the following equation:


log(*V_t_*) *= β*_0_ + *β*_1_ × *t* (1)

where log(*V_t_*) is the natural log of the average number of relative search volumes at time *t*; *β*_0_ indicates the intercept of the model; *β*_1_ is the slope of the model, which was later used to estimate the monthly percentage change of search volumes of each segment [20]; and *t* is the time variable, indicating the month (from January 2004 to November 2019, indicated as 1 to 191).

## Results

**Figure 2:**
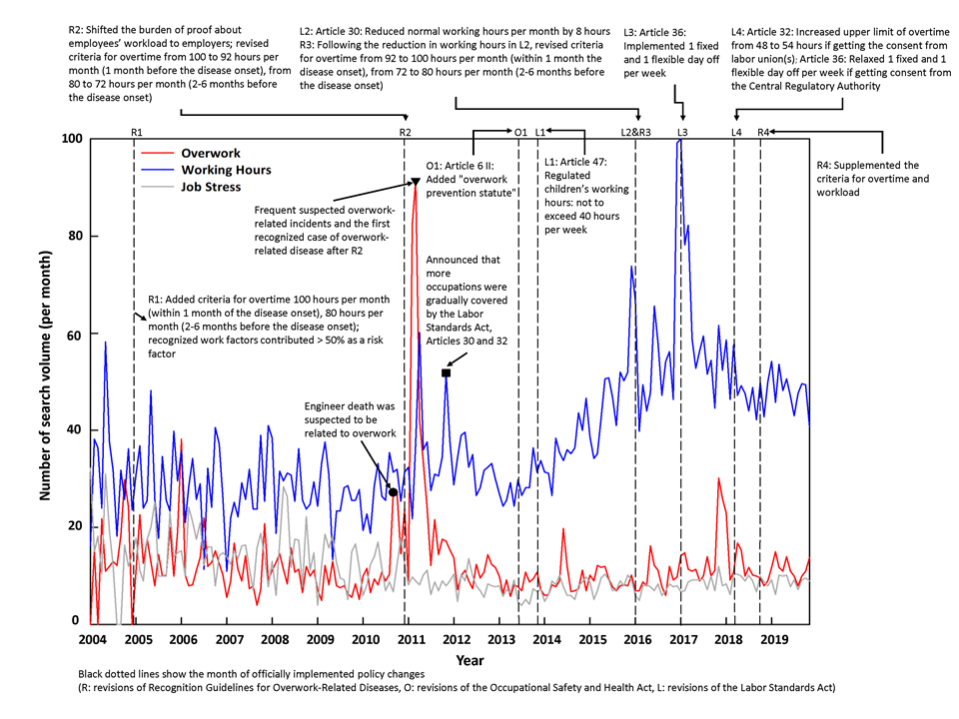
Policy changes and search volume trends for working hours, overwork, and job stress from January 2004 to November 2019.

Figure 2 shows the policy changes and the search volume trends for the keywords of the 3 working conditions. The red line shows overwork trends, the blue line shows working hours trends, and the gray line shows job stress trends. A higher search volume represents relatively higher public awareness of the topic.

Comparing the relative average search volumes of the 3 working conditions between January 2004 and November 2019, we found the highest volume was for working hours (average volumes=37), followed by overwork (average volumes=13) and job stress (average volumes=11). Most of the observed peaks occurred in 2 periods: the first from the middle of 2010 to early 2011, and the second from the middle of 2015 to early 2018. The first period included peaks in search volumes for both overwork and working hours. The first overwork peak—indicated as the filled circle on the red line—appeared in September 2010, following a death of an engineer in January 2010 that was suspected to be an overwork-related event. Then, a dramatic increase and peak for overwork appeared in March 2011—indicated as the inverted triangle on the red line, following media reports on an increase in suspected overwork-related events and the second revision of Taiwan’s Recognition Guidelines for Overwork-Related Diseases in December 2010 (dashed line R2). This phenomenon also urged the government to include more occupations (preschool educator, medical personnel, and hotel and motel bedmaker) among those covered by the Labor Standards Act Articles 30 and 32 in order to regulate their normal working hours and overtime hours. This is indicated as the filled square on the blue line in November 2011.

The second peak period appeared to be attributed mainly to policy changes related to working hours: most peaks on working hours searches matched the policy changes, such as the 12th revision of the Labor Standards Act in May 2015, and its implementation in January 2016 (dashed line L2); the 16th amendment of the Labor Standards Act, stipulating 1 fixed and 1 flexible day off per week, in December 2016, and its implementation in January 2017 (dash line L3); and the 18th amendment of the Labor Standards Act, which was implemented in March 2018 (dashed line L4). Over the entire period, the search volume for job stress remained relatively lower and more stable than those for overwork and working hours.

Table 1 presents the monthly percentage change of search volumes for the 3 working conditions, with obvious and significantly increasing trends for both overwork and working hours. From June 2010 to March 2011, the monthly percentage change of search volumes for overwork significantly increased by 28.97% per month (95% CI 19.25-39.49; *P*<.001), following (1) the frequent reports of suspected overwork-related events, especially among engineers and security personnel (Figure 2, inverted triangle), and (2) the second amendment of Recognition Guidelines for Overwork-Related Diseases in 2010 (Figure 2, line R2).

For working hours, the monthly percentage change of search volumes significantly increased from September 2013 to October 2016 by 1.78% per month (95% CI 1.14-2.43; *P*<.001). This trend occurs after the 12th amendment of the Labor Standards Act (ie, reduce 2 hours for normal working hours per week) and the disputes for 16th Labor Standards Act revision (regarding 1 fixed and 1 flexible day off per week); the search volumes peaked in concert with the implementation of the 16th amendment in January 2017.

Slightly increasing trends appeared for job stress from the middle of 2013 to the end of 2019, by 0.39% per month (95% CI 0.05-0.73; *P*=.026), but 84% of the number of search volumes per month was still lower than 10. This trend occurs after the new revision of Occupational Safety and Health Act in July 2013, which added the “overwork prevention statute.”

**Table 1:** Trends and monthly percentage change of search volumes for the 3 working conditions.

Working conditions and their periods studied		Trend (*P* value)	Monthly percentage change of search volumes (95% CI)
**Overwork**			
	January 2004 to June 2010	↓ (*P*<.001)	–1.08 (–1.45 to –0.71)
	June 2010 to March 2011	↑ (*P*<.001)	28.97 (19.25 to 39.49)
	March 2011 to July 2011	↓ (*P*=.014)	–36.06 (–55.14 to –8.87)
	July 2011 to November 2015	↓ (*P*=.004)	–0.99 (–1.66 to –0.32)
	November 2015 to December 2017	↑ (*P*=.006)	2.99 (0.86 to 5.16)
	December 2017 to November 2019	↓ (*P*=.020)	–2.50 (–4.55 to –0.41)
**Working hours**			
	January 2004 to September 2013	↔ (*P*=.768)	0.02 (–0.11 to 0.15)
	September 2013 to October 2016	↑ (*P*<.001)	1.78 (1.14 to 2.43)
	October 2016 to January 2017	↔ (*P*<.470)	19.07 (–26.00 to 91.59)
	January 2017 to May 2017	↔ (*P*=.194)	–14.22 (–32.00 to 8.22)
	May 2017 to November 2019	↔ (*P*=.121)	–0.59 (–1.34 to 0.16)
**Job stress**			
	January 2004 to January 2008	↓ (*P*<.001)	–0.93 (–1.45 to –0.41)
	January 2008 to April 2008	↔ (*P*=.810)	12.49 (–57.19 to 195.55)
	April 2008 to March 2013	↓ (*P*<.001)	–1.64 (–2.10 to –1.18)
	March 2013 November 2019	↑ (*P*=.026)	0.39 (0.05 to 0.73)

## Discussion

### Principal Findings

Over the past 15 years, Taiwanese authorities have revised and implemented several policies to prevent overwork-related health problems. By treating search volumes for overwork-related keywords as proxy indicators of public awareness, our findings suggest that increasing public awareness of the importance of overwork prevention and shorter working hours was observed after Taiwan’s recent policy changes related to overwork prevention. Such increased awareness is expected to contribute to better attitudes and practices regarding overwork prevention among the general public [9].

Public awareness of overwork significantly increased (*P*<.001) following the 2010 revision and implementation of the Recognition Guidelines for Overwork-Related Diseases, as well as the frequent media reports in 2009–2011 of suspected overwork-related incidents. Public awareness of working hours increased from 2013 and peaked twice, in the end of 2015 and early 2017, along with the two amendments (12th and 16th) of the Labor Standards Act.

### Overwork

The search volumes for overwork increased from June 2010 to March 2011, a period which saw frequent reports of suspected overwork-related incidents prompt public realization of the seriousness of the problem. During the same period, more overwork cases were recognized by the 2010 Recognition Guidelines for Overwork-Related Diseases, which encouraged public confidence in overwork-related policy. After the revised recognition policy was implemented in 2010, the average number of working hours per month of those employed in the industry and service sectors in Taiwan decreased by 9.1 hours from 178.7 hours in 2011 to 169.6 hours in 2016 [21]. The amendment to the recognition guidelines followed the sudden death, in January 2010, of an engineer in Taiwan by cardiogenic shock and hypertrophic cardiomyopathy. Although some of the disease cases could not be officially recognized as related to overwork right out, they exerted influences because a nongovernmental organization (Taiwan Labour Front) and a legislator (Ms Sue‐Ying Huang) organized several press conferences on behalf of families of deceased to promote social awareness via social media, and finally intervene in the decision making of the Ministry of Labor in Taiwan [6]. These activities had aroused public awareness about the strenuous working conditions of elite image engineers. When the 2010 Recognition Guidelines for Overwork-Related Diseases were implemented, cerebrovascular and cardiovascular diseases related to working conditions with long-term excessive overtime became the first recognized overwork disease [6]. Social media together with efforts of Legislator Huang and nongovernmental organizations appeared to have a strong influence on the policy changes in Taiwan [6,22]. Based on this understanding, one way to significantly enhance public awareness of overwork is to cooperate with legislators and nongovernmental organizations and to encourage more media reports on policy changes via social media.

### Working Hours

Public awareness about working hours significantly increased (*P*<.001) from September 2013 and reached a peak in January 2017, which included 2 relevant policy amendments to the Labor Standards Act. Among all search volume peaks in this period, the highest were observed in January 2017, aligned with the start of implementation of the 16th amendment, which called for “1 fixed and 1 flexible day off per week.” That this policy change would call forth such a high search volume might be due to presidential influence. The theme of overwork was a hot issue during Taiwan’s 2016 presidential election. Among all presidential candidates, one candidate (Dr Ing-Wen Tsai, who later won the election) was most committed to preventing overwork problem in Taiwan and even claimed to have prepared related policies that would improve working conditions. This candidate went on to get most of the labor unions’ support. Along with their candidate’s victory in the presidential election, union workers now look forward to the corresponding policy changes [23], which they expect to provide better working conditions. Since President Tsai was inaugurated in May 2016, the Labor Standards Act has been revised seven more times between November 2016 and May 2019 [24].

### Policies and Public Awareness

In our study, we observed higher search volumes around the months when policies were revised and implemented. Revision and implementation are the 2 phases wherein the public can participate in the policy change process (comprised from invention, estimation, selection [revision], implementation, evaluation, to termination) [25]. High search volume observed during policy revision and implementation periods may be attributable to the healthy democracy in Taiwan, which allows for public participation in the policy change process, promotes mutual understanding among stakeholders, and emphasizes the legitimacy of governance [26].

Public awareness on both overwork and working hours was enhanced along with the related policy changes and frequent reports of suspected overwork-related events. However, the search volume trends on overwork and working hours differ. The trend of increasing search volumes on overwork (June 2010 to March 2011) lasted for only 10 months, following the reporting of suspected overwork-related events. By contrast, the trend of increasing search volumes on working hours (September 2013 to October 2016) lasted for 38 months, following the amendments of the Labor Standards Act. Our results suggest that public awareness caused by events is short lived, whereas public awareness induced by policy exists for a longer time, likely because social media continues to provide new information and discussion on the topic [27,28]. Therefore, we suggest that developing overwork-related policies that clearly address risk factors contributes to increasing the public awareness of overwork prevention.

As a reference outcome, public awareness of job stress was lower than public awareness of overwork and working hours over the past 15 years. Given the fact that high job stress is one of the major reasons for overwork [29], we suggest that policies should also target overwork caused by job stress, similar to European policies for psychosocial risk management in the workplace [30].

### Limitations

There are some limitations to our study. First, we identified one keyword with the highest search volume as the representative keyword for each working condition. The general public might use other unpopular keywords (synonyms) to search for more knowledge on each working condition. That is, each working condition could be described using different keywords. Thus, our choice of high-volume keywords might underestimate the effect of policy changes on public awareness.

Second, we could not get an absolute value for search volume on Google Trends. Data obtained from Google Trends are a relative search volume (search volume range from 0 to 100). For cross-keyword and overtime comparisons, we could only compare search volumes for up to 5 keywords at once. Our choice of 3 keywords ensured the comparability of search volumes in our results. Future studies that attempt to expand the number of keywords compared will warrant the development of advanced adjustment methods.

Third, search volumes on Google Trends have been used to explore and predict behaviors on the outpatient visits and people’s awareness of health care [16,31-33], but have been shown to be less predictive of public opinions on elections and referendums in low-internet-use or low-freedom-of-speech regions [34]. Our study focuses on searches in Taiwan, where over 80% of the population uses the internet and people have a high degree of freedom to speak their personal opinions [35,36]. The advantages of using Google Trends should also be highlighted, including reducing the time and cost associated with the traditional measures of public awareness and acting as a supplement and complement to traditional polls or surveillance systems [37], especially in countries with high internet penetration and freedom of speech. Although Google is the most commonly used search engine in Taiwan, there are other types of search engines that may be used by different sectors of population, thus may generate different search patterns. Yet, comparative analyses of search patterns across different search engines are beyond the scope of this study. A separate study involving the collection and comparison of data from different search engines can help explore the diverse patterns.

As described in a 2014 review about big data’s features [38], the 4V concept—related to the high volume, velocity, variety, and veracity of information—was developed, along with the understanding that transforming data into value requires specific technology and analytical methods. Search volumes obtained from Google Trends are relative, and some might doubt if these search volumes should be considered big data. However, Google Trends data fit the aforesaid 4 features, and some earlier studies have set precedents for considering Google Trends data as online big data [39]. Moreover, the novel way in which Google Trends presents data largely reduces equipment requirements for big data analysis and can be used in many fields. Google Trends can in fact help government officials quickly clarify fast-changing events that raise public concern [40,41] and collect large volumes and a variety of past data in a short period, which is hard to achieve through traditional public polls.

What our study contributes to the global society, particularly labor policymakers, is that a clear policy will lead to rising public awareness, and such an increase in public awareness will reinforce policy change—leading to a virtuous circle. In addition, study findings from Taiwan, a democratic society, highlight the importance of communication capacity in informing and engaging citizens. We believe that our approach enabled a reasonable representation of countries with similar internet access, freedom of participation in political and public affairs, and government responsiveness; by contrast, countries with different political environments warrant separate analyses. We encourage the country’s citizens to have more access and freedom to engage in public affairs, particularly labor and public health issues.

### Conclusions

Overwork-related health problems due to long working hours, heavy workloads, hectic work pace, and insufficient rest time are major occupational health concerns in many East Asian countries, and in certain occupational groups of Western societies. Regulations concerning working hours, overtime pay, paid leave, and rest time have long been central issues in labor and health protection policies. While in Taiwan, the labor and health authorities have made efforts by revising laws and implementing new policies to improve work quality and to reduce overwork-related health risks, there are still controversies and debates with respect to their regulatory designs and enforcement. Analyses of substantive policies are beyond the scope of this study.

Yet, this study demonstrates rising public awareness of overwork prevention, particularly as it relates to overwork and working hours, following recent overwork-related policy changes. Public awareness of policy contents and citizens’ supports are essential for effective policy implementation. Our findings suggested that in Taiwan, certain components of overwork prevention policies were conveyed to the public more effectively while the others were conveyed less effectively. Because our findings indicate that the public are less aware of the role of job stress in overwork prevention, we suggest implementing interventions to address this phenomenon. Ultimately, to enhance public awareness of overwork prevention, national overwork-related policies that clearly address risk factors are necessary.
